# The Efficacy of Boxing Training on Patients with Parkinson's Disease: Systematic Review and Meta-Analysis 

**DOI:** 10.31083/RN36478

**Published:** 2024-12-20

**Authors:** Daniel González-Devesa, Carlos Ayán, Miguel Adriano Sanchez-Lastra, Ciro Gutiérrez-Hong, Adrián García-Fresneda, José Carlos Diz

**Affiliations:** ^1^Well-Move Research Group, Galicia Sur Health Research Institute (IIS Galicia Sur), SERGAS-UVIGO, 36310 Vigo, Spain; ^2^Grupo de Investigación en Actividad Física, Educación, y Salud (GIAFES), Universidad Católica de Ávila, C/ Canteros, 05005, Ávila, Spain; ^3^Departamento de Didácticas Especiais, Universidad de Vigo, 36310 Vigo, Spain; ^4^Research Group in Tecnologia Aplicada a l’Alt Rendiment i la Salut (TAARS), TecnoCampus, Department of Health Sciences, Pompeu Fabra University, 08302 Mataró, Spain

**Keywords:** balance, exercise, physical activity, rehabilitation, equilibrio, ejercicio, actividad física, rehabilitación

## Abstract

**Objective:**

This study aimed to systematically review the available evidence on the effects of boxing interventions on people with Parkinson disease.

**Methods:**

Four electronic databases were searched systematically from their inception until December 2023. The methodological quality of the included studies was assessed using the Physiotherapy Evidence Database and Methodological Index for Non-Randomized Studies scales.

**Results:**

A total of 13 studies were included. Data synthesis indicated that participants who performed boxing programs did not have a significant effect in the polled data on functional mobility, balance, motor symptoms, gait and cardiorespiratory fitness Accordingly, for the effects of boxing on self-reported quality of life, the polled data showed a non-significant trend towards improving Hedges' g. Also, when the analysis was performed by comparing the experimental and control groups, the results remained non-significant.

**Conclusions:**

The evidence regarding the use of boxing as a program exercise for patients with Parkinson disease remains uncertain. Preliminary findings indicate that participation in boxing does not demonstrate a substantial impact on either physical or mental health outcomes.

## Abstract (es)

**Objetivo:**

Este estudio tuvo como objetivo revisar sistemáticamente la evidencia disponible sobre los efectos de las intervenciones de boxeo en personas con enfermedad de Parkinson.

**Métodos:**

Se realizaron búsquedas sistemáticas en cuatro bases de datos electrónicas desde su creación hasta diciembre de 2023. La calidad metodológica de los estudios incluidos fue evaluada utilizando las escalas Physiotherapy Evidence Database (PEDro) y Methodological Index for Non-Randomized Studies.

**Resultados:**

Se incluyeron un total de 13 estudios. La síntesis de los datos indicó que los participantes que realizaron programas de boxeo no mostraron un efecto significativo en los datos agrupados sobre movilidad funcional, equilibrio, síntomas motores, marcha y capacidad cardiorrespiratoria. Asimismo, en cuanto a los efectos del boxeo en la calidad de vida autoinformada, los datos agrupados mostraron una tendencia no significativa hacia la mejora según el estadístico Hedges' g. Además, cuando se realizó el análisis comparando los grupos experimentales y de control, los resultados permanecieron no significativos.

**Conclusiones:**

La evidencia sobre el uso del boxeo como programa de ejercicio para pacientes con enfermedad de Parkinson sigue siendo incierta. Los hallazgos preliminares indican que la participación en boxeo no demuestra un impacto sustancial en los resultados de salud física o mental.

## 1. Introduction

Parkinson’s disease (PD) is a complex neurodegenerative condition that greatly impacts daily life and well-being. With no available disease-modifying treatments, rehabilitation plays a crucial role in managing the disease [1]. Among rehabilitation approaches, physical exercise stands out as key in delaying PD progression [2]. Reviews and meta-analyses have shown the benefits of various exercise types, such as aerobic, resistance training, and alternative activities like dancing or Pilates, on mobility and quality of life in PD patients [3]. However, patients often face barriers to maintaining regular exercise, making enjoyment a key factor in adherence [4].

In this context, boxing has emerged as a promising alternative. Studies show it is more enjoyable than traditional exercises for stroke patients and leads to significant improvements in functional mobility, balance, gait, and quality of life [5, 6]. Boxing has also been linked to better mental health and increased motivation for physical activity in PD patients [7, 8].

While boxing programs are gaining popularity among PD patients, there is limited research on their impact [9]. Health professionals should carefully consider the potential benefits and risks of boxing therapy. To date, only one narrative review and one systematic review have examined the effects of boxing on PD [10, 11].

Given the growing interest, this study aims to conduct a systematic review and meta-analysis to critically assess the available evidence on boxing as a rehabilitation therapy for people with PD.

## 2. Material and Methods

This systematic review was conducted in accordance with the Preferred Reporting Items for Systematic Reviews and Meta-Analyses (PRISMA) guidelines, the checklist see the **Supplementary Materials** [12].

### 2.1 Search Strategy

A systematic search was conducted in four electronic databases (MEDLINE/PubMed, Web of Science, SPORTDiscuss and Scopus) covering the period from their inception until March 2024. The following search terms, Boolean operators, and combinations were used: (“Parkinson” OR “Parkinson disease”) AND (“Boxing” OR “Boxing therapy” OR “Boxing program” OR “Boxing exercise” OR “Counterpunching” OR “Non-contact boxing” OR “Rock Steady Boxing”).

### 2.2 Eligibility Criteria

Eligible studies for inclusion in this review were investigations published in peer reviewed journals that provided information on the effects of boxing-based interventions on individuals with PD. The following criteria were used to exclude investigations from the review: (a) studies where boxing was combined with other exercise training modalities; (b) studies that included individuals with multiple pathologies, unless separate data were available specifically for the PD subgroup; (c) case studies; (d) qualitative studies; (e) studies that were not written in English, Portuguese, or Spanish language, and (f) studies for which the full text was not available.

### 2.3 Study Selection

Two authors independently screened the titles and abstracts of the identified studies to determine their eligibility. Following this initial screening, the selected studies were reviewed by both authors to assess their suitability for inclusion. Any discrepancies or disagreements were discussed and resolved through mutual consensus. Subsequently, full-text copies of potentially relevant studies were obtained. In cases where there was uncertainty regarding whether a study met the selection criteria, a third author was consulted to provide input, and a consensus was reached through discussion. The reference lists of the articles selected, as well studies that quoted them in Google Scholar, were checked for potential new articles eligible for this review.

### 2.4 Data Extraction

Information on participants’ characteristics, boxing interventions, variables assessed, main outcomes, adverse events and drop-outs were extracted by one researcher from the original reports and then cross-checked by a second investigator. Missing data were obtained from the study authors, whenever possible.

### 2.5 Quality Appraisal

The methodological quality of each randomized controlled trial (RCT) was retrieved from the Physiotherapy Evidence Database (PEDro). If a trial was not included in PEDro, two authors independently assessed its quality. Any disagreements between the authors were resolved through discussion and consensus. The quality of the studies were categorized according to the following cut-off points: excellent (9–10), good (6–8), fair (4–5), and poor (<3) [13].

The methodological quality of comparative and single arms studies were assessed by means of the Mixed Methods Appraisal Tool (MMAT) [14]. Two reviewers independently assessed the investigations using a ‘yes’, ‘can’t tell’ and ‘no’ format. Calculating an overall score is discouraged.

### 2.6 Meta-Analysis

We performed the meta-analysis calculations in Microsoft Excel with Meta-Essentials Workbooks [15], using the Hedges’ g to calculate the effect size for quantitative dependent variables. We selected the random effects model for all analysis to account for the sources of heterogeneity among different studies, using the inverse variance method.

The I^2^ was applied to assess statistical heterogeneity and inconsistency. In addition to 95% confidence intervals (CI), we calculated the prediction intervals to express both the magnitude and consistency of the effects. The forest plot was used to summarize the findings. Funnel plot and Egger’s test were used to evaluate statistically the presence of any publication bias. The trim-and-fill analysis was also included for the adjustment of potentially missing studies.

## 3. Results

### 3.1 Study Selection 

**Fig. 1.**
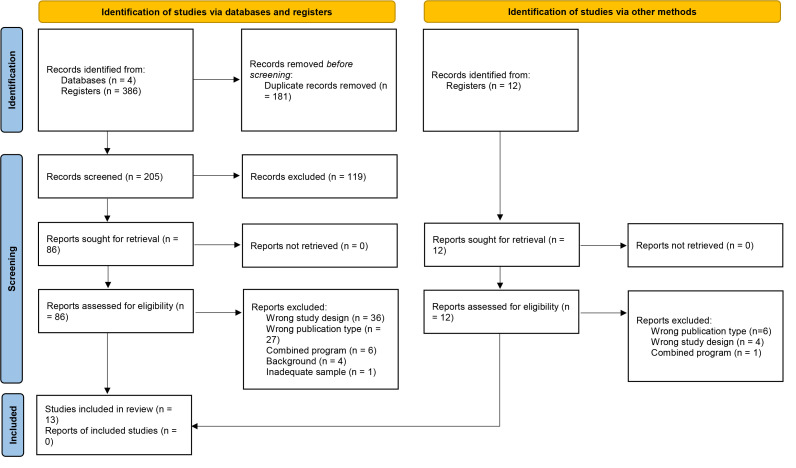
**Flow diagram of the search and selection process for the inclusion of articles**.

Of the 386 records initially obtained, 13 studies met the inclusion criteria for this systematic review (Fig. 1).

### 3.2 Study Characteristics

Among the 13 selected studies, four included a comparison group. Three were randomized controlled trials comparing two types of boxing programs [16], boxing versus sensory training [17] and boxing versus traditional exercise [18]. The non-randomized study by Salvatore *et al*. [19] did not conduct an intervention in the comparison group. Several studies were single-arm trials [20, 21, 22], with others categorized as retrospective (n = 4) [23, 24, 25, 26], prospective observational (n = 1) [27], and case series (n = 1) [28]. All studies were published between 2011 and 2023. The total sample size was 423 participants, ranging from 6 to 98 patients. Participants varied widely in age (31 to 90 years) and Hoehn and Yahr stages (1 to 4), with a majority being male (72%); gender data was missing in two studies [17, 28]. Eight studies included participants on standard PD medication, while six tested in the ON-medication state [17, 19, 21, 27, 28].

### 3.3 Boxing Intervention Characteristics 

Nine studies classified the intervention as non-contact boxing [17, 18, 19, 20, 21, 23, 24, 25, 28], while others used terms like high-intensity boxing, boxing therapy, or community-based boxing program [16, 22, 26]. Exercise interventions lasted from 10 weeks to 24 months, with 1 to 4 sessions per week. Session duration varied from 45 to 90 minutes (Table 1, Ref. [16, 17, 18, 19, 20, 21, 22, 23, 24, 25, 26, 27, 28]).

**Table 1.** **Descriptive characteristics of the included studies**.

First Author (Year), Design and Country	Sample	Intervention	Outcomes	Results	Dropouts, Adverse Events and Adherence
Domingos *et al*. (2022) [16]	**Participants (n):**	**Duration:** 10-weeks	**Functional Mobility:**	**Intra-group *(p < 0.05)***	**Dropouts:** 4;
	29 (EG1 = 14; EG2 = 15);	**EG1**	∙ TUG (s)	↓ TUG performance in IG1 after intervention (7.74 ± 2.21 vs 8.88 ± 2.36 s)	Did not receive allocated intervention (n = 1), Moved to another city (n = 1), Hospitalization (n = 2)
**Design:** RCT	EG1: 6M + 8F; EG2: 8M+7F	**Type:** Boxing	∙ dTUG (s)		
**Country:**	**Age, *years* (mean; SD):** EG1 = 64.36 ± 11.14; EG2 = 63.69 ± 6.63	**Activities:** Training punching movements and progressively adding in dual task challenges (i.e., going through a variety of punching combinations)	**Quality of life:**	↓ PDQ-39 score in IG1 after intervention (26.26 ± 18.08 vs 19.01 ± 10.62)	
The Netherlands	**Disease duration, *years* (mean; SD): **EG1 = 6.10 ± 4.72; EG2 = 9.09 ± 5.73	**Volume:** 60 min/session	∙ PDQ-39 (score)	↑ Mini-BESTest performance in IG1 after intervention (23.09 ± 3.44 vs 25.80 ± 2.39)	**Adverse events:** None
		**Frequency:** 1 days/week	Balance:		**Adherence: **92.86%
	**Hoehn and Yahr: **NR	**Intensity:** NR	∙ Mini-BESTest	↑ Mini-BESTest performance in IG2 after intervention (22.60 ± 2.70 vs 25.33 ± 2.64)	Intention-to-treat
	**Dopamine replacement:**	**EG2**	∙ ABC-scale (score)		
	EG1 = 10/14; EG2 = 13/15	**Type:** Boxing with kicking techniques	**Cardiorespiratory fitness**	**Inter-group (p < 0.05)**- NO	
		**Activities:** Boxing training comparable to the	∙ 6MWD (m)		
		previous group, but with added kicking techniques, weight shifting exercises and multidirectional stepping.	**Fear of falling:**		
			∙ FES-I (score)		
		**Volume:** 60 min/session			
		**Frequency:** 1 days/week			
		**Intensity:** NR			
Sangarapillai *et al*. (2021) [17]	**Participants (n):**	**Duration:** 20-weeks (10-weeks intervention + 10-weeks washout)	**Quality of life:**	**Intra-group *(p < 0.05)***	**Dropouts:** None
	40 (EG1 = 20; EG2 = 20)		∙ PDQ-39 (score)	↑ UPDRS-III scores in IG1 after intervention and after washout (28.37 ± 11.22 vs 33.41 ± 9.67 and 34.14 ± 10.69, respectively)	**Adverse events:** None
**Design:** RCT	**Age, *years* (mean; SD):** EG1 = 64.2 ± 9.8; EG2 = 65.1 ± 9.2	**EG1**	**Motor symptoms:**		**Adherence: **100%
**Country:**		**Type:** Rock Steady Boxing	∙ UPDRS-III (score)		
	**Disease duration, *years* (mean; SD): **EG1 = 6.38 ± 4.9; EG2 = 7.82 ± 5.2	**Activities:** Warm-up, boxing specific exercise (high intensity boxing drills, shadow boxing, jumping jacks, speedbag drills) and cool down		↓ UPDRS-III scores in IG2 after intervention and after washout (28.8 ± 10.19 vs 19.6 ± 10.03 and 20.45 ± 10.95, respectively)	
				↓ Stride length in IG1 after washout (1.48 ± 0.24 vs 1.39 ± 0.22 m)	
Canada	Hoehn and Yahr (mean):	**Volume:** 60 min/session	**Gait:**	↑ Stride length in IG2 after intervention and after washout (1.46 ± 0.13 vs 1.73 ± 0.52 and 1.76 ± 0.38 m, respectively)	
	EG1 = 2.5; EG2 = 2.5	**Frequency:** 3 days/week	∙ Stride length (m)		
	**Dopamine replacement:** ON/ Levodopa dose (mg/d)	**Intensity:** NR	∙ Stride velocity (m/s)		
		**EG2**		↓ Stride velocity in IG1 after washout (1.4 ± 0.17 vs 1.32 ± 1.18 m/s)	
	EG1: 612.13 ± 220.75	**Type:** Sensory attention focused training			
	EG2: 608.11 ± 238.44	**Activities:** Warm-up, sensory exercises (including stretches, walking, and chair exercises performed slowly and with eyes closed), and a cool down.		↑ Stride length in IG2 after washout (1.43 ± 0.13 vs 1.55 ± 0.21 m/s)	
				**Inter-group *(p < 0.05)***	
		**Volume:** 60 min/session		> Improvements in UPDRS-III scores on EG2 compared with EG1 after intervention (9.2 ± 1.1 vs –5.04 ± 0.8) and after washout (8.35 ± 1.2 vs –5.77 ± 0.9)	
		**Frequency:** 3 days/week			
		**Intensity:** NR			
				>Stride length in EG2 compared with EG1 after intervention (1.73 ± 0.52 vs 1.47 ± 0.22m) and after washout (1.76 ± 0.38 vs 1.39 ± 0.22 m)	
				>Stride velocity in EG2 compared with EG1 after intervention (1.53 ± 0.2 vs 1.36 ± 0.18 m/s) and after washout (1.55 ± 0.21 vs 1.32 ± 1.18 m/s)	
Combs *et al*. (2013) [18]	**Participants (n):**	**Duration:** 12-weeks	**Functional Mobility:**	**Intra-group *(p < 0.05)***	**Dropouts:** 8; Withdrew schedule conflict (n = 2), Changed health status (n = 2),
**Design:** RCT	31 (EG1 = 17; EG2 = 14);	**EG1**	∙ TUG (s)	↑ TUG performance in IG1 after intervention (8.05 ± 15.12 vs 7.12 ± 14.62 s)	
**Country:**	EG1: 11M+6F; EG2: 10M + 4F	**Type:** Rock Steady Boxing	∙ dTUG (s)		
USA	**Age, *years* (median; range):** EG1 = 66.5 (28); EG2 = 68 (31)	**Activities:** Warm-up, of boxing specific activities via a circuit training regimen, as well as general endurance activities.	**Quality of life**	↑ TUG performance in IG2 after intervention (7.64 ± 7.39 vs 7.12 ± 5.47 s)	Did not like assigned program (n = 2),
	**Disease duration, *months* (median; range): **EG1 = 41.5 (182); EG2 = 50 (99)		∙ PDQL (score)	↑ dTUG performance in IG1 after intervention (11.32 ± 26.23 vs 8.16 ± 18.24 s)	Did not complete minimum number of training sessions (n = 2)
		**Volume:** 90 min/session	**Balance:**		
	**Hoehn and Yahr (median; range):** EG1 = 2 (3); EG2 = 2 (3)	**Frequency:** 2–3 days/week		↑ dTUG performance in IG2 after intervention (10.33 ± 16.09 vs 8.89 ± 7.64 s)	**Adverse events:** None
					**Adherence: **88.24%
				↑ PDQL score in IG1 after intervention (128 ± 61 vs 132 ± 63)	Intention-to-treat; carried last score forward for those lost to follow upIntention-to-treat; carried last score forward for those lost to follow up
	Dopamine replacement: ON	**Intensity:** NR	∙ BBS (score)	↑ PDQL score in IG2 after intervention (125.5 ± 84 vs 149.5 ± 79)	
		**EG2**	∙ ABC-scale (%)		
		**Type:** Traditional exercise	**Gait:**	↑ BBS score in IG1 after intervention (49 ± 49 vs 53 ± 45)	
		**Activities:** Warm-up, strengthening exercises, endurance training, balance activities and a cool down.	∙ Gait velocity (m/s)		
			**Cardiorespiratory fitness**	↑ BBS score in IG2 after intervention (49 ± 17 vs 54 ± 12)	
		**Volume:** 90 min/session	∙ 6MWD (m)	↑ ABC-scale score in IG2 after intervention (85 ± 56.9 vs 93.3 ± 33.8 %)	
		**Frequency:** 2–3 days/week			
		**Intensity:** NR		↑ Gait velocity in IG1 after intervention (1.06 ± 1.08 vs 1.10 ± 1.10 m/s)	
				↑ 6MWT performance in IG1 after intervention (405 ± 549.1 vs 457 ± 669.7 m)	
				**Inter-group *(p < 0.05*)**	
				< ABC-scale score in EG1 compared with EG2 after intervention (85.3 ± 60.6 vs 93.3 ± 33.8 %)	
Salvatore *et al*. (2022) [19]	**Participants (n): **45	**Duration:** ≥3 months	**Functional Mobility:**	**Inter-group (*p* < 0.05)**	**Dropouts:** 2
	EG: 21 (12M + 9F);	**EG**	∙ TUG (s)	> TUG performance in EG compared with CON (7.31 ± 1.52 vs 9.40 ± 3.44 s)	**Adverse events:** NR
**Design:**	CON: 24 (17M + 7F)	**Type:** Non-contact Boxing	**Cardiorespiratory fitness**		**Adherence: **NR
Comparative	**Age, *years* (mean; SD):**	**Activities:** Warm-up (10-15 min), ∼30 min of supervised continuous rotations alternating through 7 stations (e.g., speed bag, heavy bag, footwork, trainer mit dodging, bobbing, etc), then completed 10-15 min resistance training with a cool-down.		> Trail–making Test A performance in EG compared with CON (36.82 ± 10.35 vs 46.23 ± 14.48)	
**Country:**	EG = 68.71 ± 7.15		∙ 6MWD (m)		
USA	CON = 71.33 ± 8.56		**Executive function:**		
	**Disease duration, *years*:**		∙ Trail–making Test A and B		
	EG = 68.71 ± 7.15				
	CON = 71.33 ± 8.56	**Volume:** 50–60 min/session			
	**Hoehn and Yahr: ≤**1.5	**Frequency:** ≥3 days/week			
	**Dopamine replacement:**	**Intensity:** ∼67% HR_max_ (moderate)			
	ON/ Levodopa dose (mg/d)	**CON**: Non-exercising			
	561.55 ± 425.53				
Blacker *et al*. (2023) [20]	**Participants (n):**	**Duration:** 15-weeks	**Motor symptoms:**	**Intra-group *(p < 0.05)-*** NO	**Dropouts:** None
**Design:**	10; 6M + 4F	**Type:** Non-contact Boxing	∙ UPDRS-III (score)		**Adverse events:** 5; Hospitalized due to an overdose of
Single-arm	Age, *years* (mean; SD):	**Activities:** Warm-up (10 min), boxing-specific movements and aerobics (10 min), boxing sequences (25 min)			
**Country:**	59.07 ± 7.38				medications and alcohol (n = 1)
Australia	**Disease duration, *years* (mean; SD): **4.08 ± 3.12				Strained a calf muscle (n = 1), Exacerbated a preexisting knee injury (n = 1),
		**Volume:** 45 min/session			
	**Hoehn and Yahr:**	**Frequency:** 3 days/week			
	Score 1 (n = 8), Score 2 (n = 2)	**Intensity:**			Foot pain (n = 2)
	**Dopamine replacement:**	1–5 week: 70% HR_max_, <7 RPME;			**Adherence: **96.7%
	9/10	6–10 week: >80% HR_max_, <7 RPME;			
		11–15 week: 70–80% HR_max_, >7 RPME			
Watts *et al* (2023) [21]	**Participants (n): **8M	**Duration:** 12-months	**Respiratory and Phonation:**	**Intra-group *(p < 0.05)***	**Dropouts:** None
**Design:**	**Age, *years* (mean; SD):**	**Type:** Non-contact Boxing		↑ Maximum expiratory pressure at the 9-month period and at the 12-month (63.25 ± 36.7 vs 101.99 ± 45.12 and 115.25 ± 49.27 cmH_2_O, respectively)	**Adverse events:** NR
Single-arm	67.08 ± 6.89	**Activities:** Warm-up (10 min), continuous rotations alternating through 7 stations (e.g., speedbag, heavy bag, footwork, hand mitts, dodging, and bobbing, etc.) during ∼30 min, followed by 10–15 min resistance training and cool-down.	∙ Maximum expiratory pressure (cmH_2_O)		**Adherence: **NR
**Country:**	**Disease duration, *years* (range): **1-15				
USA	**Hoehn and Yahr:**		∙ Transglottal Airflow (L/s)		
	Score 1 = 3, Score 2 = 1, Score 3 = 4				
	**Dopamine replacement:**		∙ Subglottal Pressure (cmH_2_O)		
	ON	**Volume:** 60 min/session			
		**Frequency:** 2 days/week			
		**Intensity:** NR			
Urrutia *et al*. (2020) [22]	**Participants (n):**	**Duration:** 12-weeks (6-weeks intervention + 6-weeks follow-up)	**Quality of life**	**Intra-group *(p < 0.05)***	**Dropouts:** 4; Medical reasons not related to the study (n = 3), Loss of interest (n = 1)
**Design:**	15; 10M + 5F		∙ ESS (score)	↓ HDS score at the 6-weeks and at the 12-weeks (11.7 ± 4.3 vs 8.3 ± 3 and 7 ± 3.4, respectively)	
Single-arm	**Age, *years* (mean; SD):**	**Type:** High-intensity Boxing	**Depression**		
**Country:**	66.1 ± 8.9	**Activities:** Warm-up (10 min), High-intensity boxing session (e.g., combinations, heavy bag, and focus mitt drills) during 30 min and cool-down (10 min).	∙ HDS (score)		**Adverse events:** NR
USA	**Disease duration, *years* (mean; SD): **5.5 ± 4.8		**Sleep Disturbances**	↑ PDSS score at the 6-weeks (95.8 ± 18.3 vs 109.4 ± 15.4)	**Adherence: **NR
			∙ PDSS (score)		
	**Hoehn and Yahr (mean; SD, range): **1.6 ± 0.91, 1–3				
		**Volume:** 50 min/session			
	Dopamine replacement:	**Frequency:** 2 days/week			
	NR	**Intensity:** 80–85% HR_max_ during boxing session			
Patel *et al*. (2023) [27]	**Participants (n):**	**Duration:** 12-weeks	**Quality of life**	**Intra-group *(p < 0.05)***	**Dropouts:** n = 8;
**Design:**	14; 8M + 6F	**Type:** Community-based boxing program	∙ PDQ-39 (score)	↓ MDS-NMS pain subscale post-intervention (10.7 ± 6.6 vs 5.4 ± 5.3)	Due to scheduling conflict (n = 1),
Prospective Observational	**Age, *years* (mean; SD):**	**Activities:** Footwork, shifting, strikes/ punches, and combination strikes. Jump rope techniques were taught, including the swing and motion of jump roping. Mental performance concepts were also incorporated breathing techniques, visualisation and mental rehearsal, goal setting and self-talk.	∙ MDS-NMS (score)		Due to non- Parkinson related medical reasons (n = 1)
	62.2 ± 9		■ Apathy	↓ MDS-NMS other subscale post-intervention (17.8 ± 10.8 vs 12.4 ± 9.7)	
**Country:**	**Disease duration, *years* (mean; SD):**		■ Cognitive		Due to Parkinson-related balance problems which limited participation (n = 1),
USA	7.9 ± 4.4		■ Orthostatic hypotension	↓ HDS score post-intervention (6.3 ± 4.4 vs 3.6 ± 2.6)	
	**Hoehn and Yahr (mean; SD): **2				
	**Dopamine replacement:**		■ Urinary	↓ MDS-NMS depression subscale score post-intervention (7.9 ± 10.3 vs 3.4 ± 5.8)	Did not complete the post-assessment (n = 5)
	ON/ 12/14	**Volume:** 60 min/session	■ Sexual		
		**Frequency:** 2 days/week	■ Gastrointestinal	↓ UPDRS-III-mod score post-intervention (17.6 ± 6.4 vs 14.6 ± 5.7)	**Adverse events:** NR
		**Intensity:** NR	■ Sleep and Wakefulness		**Adherence: **NR
			■ Pain		
			■ Other		
			∙ HDS (score)		
			∙ LARS (score)		
			∙ SE-ADL (score)		
			**Depression**		
			∙ MDS-NMS (score)		
			■ Depression		
			■ Anxiety		
			**Motor symptoms:**		
			∙ UPDRS-III-mod (score)		
Horbinski *et al*. (2021) [26]	**Participants (n):**	**Duration (mean; SD, range):**	**Functional Mobility:**	**Intra-group *(p < 0.05)***	**Dropouts:** NR
	98; 76M + 22F	Pre-lockdown: 16 ± 7.98 (2-50) months	∙ Number stands from a chair in 15 s.	↑ Number of times were able to stand upright from a sitting position in a 15s during pre-lockdown and post-lockdown.	**Adverse events:** NR
**Design:**	**Age, *years* (mean; SD):**	Covid-19 lockdown: 3.22 ± 0.7 (3–7) months			**Adherence: **NR
Retrospective Observational	70.6 ± 7.98	Post-lockdown: 4.73 ± 1 (1–10) months	∙ Walk normally.		
	**Disease duration, *years*: **NR	**Type:** Boxing Therapy	∙ Heel-toe touch.	↑ Relative risk of falling from the beginning of the lockdown to the resumption of the intervention (51% each month)	
**Country:**	**Hoehn and Yahr: **NR	**Activities:** The program, with hundreds of exercises, offers three phases: (1) Stance and Posture: Master the basic “set position” for balance and stability. (2) Footwork: Develop agility with forward, side, and backward steps based on the stance. (3) Punching: Learn and practice punches for maximum force, timed with proper balance and footwork. Each phase must be mastered before progressing.	∙ Crossover		
USA	**Dopamine replacement:**		∙ Walk backwards		
	NR		∙ Stand from the floor.	↓ Relative risk of falling post-lockdown (20% each month)	
			∙ Number of falls		
			**Balance**	↑ Stand on each leg for 30s post-lockdown for both legs (5–7%)	
			∙ Stand on each leg for 30 s		
		**Volume:** NR			
		**Frequency:** NR			
		**Intensity:** NR			
Sonne *et al*. (2021) [25]	**Participants (n):**	**Duration:** 24-months	**Functional Mobility:**	**Intra-group *(p < 0.05)***	**Dropouts:**
**Design:**	68; 54M+12F	**Type:** Rock Steady Boxing	∙ 30CST (rep)	↑ 30CST performance at 6-month (19.4 ± 4.3%) and at 12-month (25.2 ± 5.9%)	Baseline
Retrospective	**Age, *years* (mean; SD):**	**Activities:** Include aerobic training with walking or jumping ropes, resistance exercises, agility exercises, non-contact boxing with the use of punching gloves and punching bags, hand–eye coordination or manual dexterity activities and stretching	∙ TUG (s)		(sample = 68),
Multicenter **Country:**	71.2 ± 8.56		**Balance:**	↑ TUG at 6-month (7.2 ± 3.4%) and at 12-month (13.7 ± 3.2%)	6 months
	**Disease duration, *years*: **NR		∙ FAB (score)		(sample = 50),
USA	**Hoehn and Yahr: **NR		**Gait**	↑ FAB score at 6-month (3.4 ± 1.5%), at 12-month (9.7 ± 1.9%) and at 18 month (11 ± 2.7%)	12 months (sample = 42),
	**Dopamine replacement:**		∙ 10MWT		18 months (sample = 17),
	NR	**Volume:** 90 min/session			24 months (sample = 8)
		**Frequency:** 2-3 days/week			**Adverse events:** NR
		**Intensity:** NR			**Adherence: **NR
Moore *et al*. (2021) [24]	**Participants (n):**	**Duration (mean; SD):** 6.1 ± 0.8 months	**Functional Mobility:**	**Intra-group *(p < 0.05)***	**Dropouts:** NR
**Design:**	12; 9M+3F	**Type:** Rock Steady Boxing	∙ TUG (s)	↑ TUG performance after intervention (8.2 ± 1.8 vs 7.3 ± 1.7s)	**Adverse events:** NR
Retrospective	**Age, *years* (mean; SD):**	**Activities:** Warm-up (15 min), boxing drills (30 min), strength and endurance exercises (15 min), activities focused on fine motor skills (15 min) and cool-down (15 min)	**Balance:**		**Adherence: **NR
Cross-sectional	67.6 ± 6.1		∙ FAB (score)	↑ FAB score after intervention (33.8 ± 4.3 vs 36.3 ± 2.6)	
**Country:**	**Disease duration, *years*: **NR				
USA	**Hoehn and Yahr (range): **1–3				
	NR	**Volume:** 90 min/session			
	**Dopamine replacement:**	**Frequency (mean; SD):** 2.8± 0.8 days/week			
		**Intensity:** 13-17 RPE			
Dawson *et al*. (2020) [23]	**Participants (n):**	**Duration:** 16-weeks	**Functional Mobility:**	**Intra-group *(p < 0.05)***	**Dropouts:** Missing data (n = 15)
**Design:**	47; 34M + 12F	**Type:** Rock Steady Boxing	∙ 30CST (rep)	↑ 30CST performance after intervention	
Retrospective	**Age, *years* (mean; SD):**	**Activities:** Warm-up (30 min), circuit training regimen (e.g., cardio, agility, boxing, calisthenics, hand-eye coordination, and strength conditioning) during 45 min and cool-down (15 min).	∙ TUG (s)	↑ TUG performance after intervention	**Adverse events:** NR
Observational	68.3 ± 7.5		**Quality of life**	↑ Pain after intervention	**Adherence: **NR
**Country:**	**Disease duration, *years *(mean; SD):** 4.24 ± 4.55		∙ EQ-5D (score)	↓ Subjective body stiffness after intervention	
USA			■ Mobility		
	**Hoehn and Yahr: **NR	**Volume:** 90 min/session	■ Self-Care	↑ Subjective mood after intervention	
	**Dopamine replacement:**	**Frequency (mean; SD):** 4 days/week	■ Usual Activities	↓ Subjective fatigue after intervention	
	NR	**Intensity:** NR	■ Pain	↑ Subjective gait and balance after intervention	
			■ Anxiety		
			■ Health State		
			∙ Subjective changes		
			■ Tremors		
			■ Body stiffness		
			■ Mood		
			■ Fatigue		
			■ Gait and balance		
Combs *et al*. (2011) [28]	**Participants (n): **6	**Duration:** 12-weeks (with the option of continuing the training for an additional 24 weeks)	**Motor symptoms:**	↓ UPDRS score at 12-, 24-, and 36-week.	**Dropouts:** Medical reasons not related to the study (n = 1)
**Design:**	**Age, *years* (mean; SD):**		∙ UPDRS-III (score)	↑ TUG performance at 12-, 24-, and 36-week.	
Case-Series	60.17 ± 10.26	**Type:** Rock Steady Boxing	**Functional Mobility:**		**Adverse events:** NR
**Country:**	**Disease duration, *months* (mean; SD):**	**Activities:** Warm-up (20 min), circuit training regimen (e.g., functional training, endurance training, punching activities and focus mitts) during 45–60 min and cool-down (15–20 min).	∙ TUG (s)	↑ PDQL score at 12-, 24-, and 36-week.	**Adherence: **NR
USA	28.67 ± 24.34		**Quality of life**	↑ BBS score at 12-, 24-, and 36-week.	
	**Hoehn and Yahr (mean; SD, range):**		∙ PDQL (score)	↑ Functional Reach Test performance at 24-week.	
	2.17 ± 1.33 (1–4)		∙ UPDRS (score)		
	**Dopamine replacement:**	**Volume:** 90 min/session	**Balance:**	↑ ABC score at 12- and 24-week.	
	4/6, ON	**Frequency:** 2–3 days/week	∙ BBS (score)	↑ Gait velocity at 24- and 36-week.	
		**Intensity:** NR	∙ Functional Reach Test	↑ Cadence at 12-, 24-, and 36-week.	
			∙ ABC-scale (%)	↑ Stride length at 12-, 24-, and 36-week.	
			**Gait:**	↓ Step width at 12-, 24-, and 36-week.	
			∙ Gait velocity (m/s)	↑ 6MWD distance at 12- and 24-week.	
			∙ Cadence		
			∙ Stride length (m)		
			∙ Stride velocity (m/s)		
			∙ Step width		
			**Cardiorespiratory fitness**		
			∙ 6MWD (m)		

>, Greater; <, Lower; ↑, Increment; ↓, Decrement; 6MWD, 6-minute walk distance; 10MWT, 10 Meter Walk Test; 30CST, 30-second Chair Stand; ABC-scale, Activities Balance Confidence Scale; BBS, Berg Balance Scale; CON, Control Group; dTUG, Dual-task Timed-Up-Go; EG, Experimental Group; EQ-5D, Euroquol-5D; ESS, Epworth Sleepiness Scale; FAB, Fullerton Advanced Balance; HDS, Hamilton Depression Scale; HR, Heart rate; F, Female; FES-I, Falls Efficacy Scale International; IG, Intervention Group; LARS, Lilli Apathy Rating Scale; M, Male; MDS-NMS, Movement Disorders Society Non-Motor Rating Scale; Mini-BESTest, Mini-Balance Evaluation Systems; NO, Not Observed; NR, Not Reported; PDQL, Parkinson’s disease Quality of Life scale; PDQ-39, Parkinson’s Disease Questionnaire; PDSS, Parkinson’s Disease Sleep Scale; RPE, Rate of Perceived Exertion; RPME, Rate of Perceived Mental Exertion; SE-ADL, Schwab and England Activities of Daily Living scale; TUG, Timed-Up-Go; UPDRS-III, Unified Parkinson’s Disease Rating Scale (motor subsection); RCT, randomized controlled trial; ON, Being under treatment during the intervention.

## 4. Main Outcomes 

### 4.1 Physical Function

#### 4.1.1 Functional Mobility 

Eight studies analyzed the effects of boxing on functional mobility, with all but one reporting significant improvements [16, 18, 19, 23, 24, 25, 28]. Although boxing was not superior to traditional exercise, it was more effective than a usual lifestyle. The Timed Up and Go test (TUG) was used in seven studies [16, 18, 23, 24, 25, 28], revealing no significant effects from boxing (Hedges’ g –0.35; 95% CI –0.94; 0.23, *p* = 0.139), with high homogeneity (I^2^ = 97.44%) and no publication bias (Egger’s *p* = 0.681).

#### 4.1.2 Balance

**Fig. 2.**
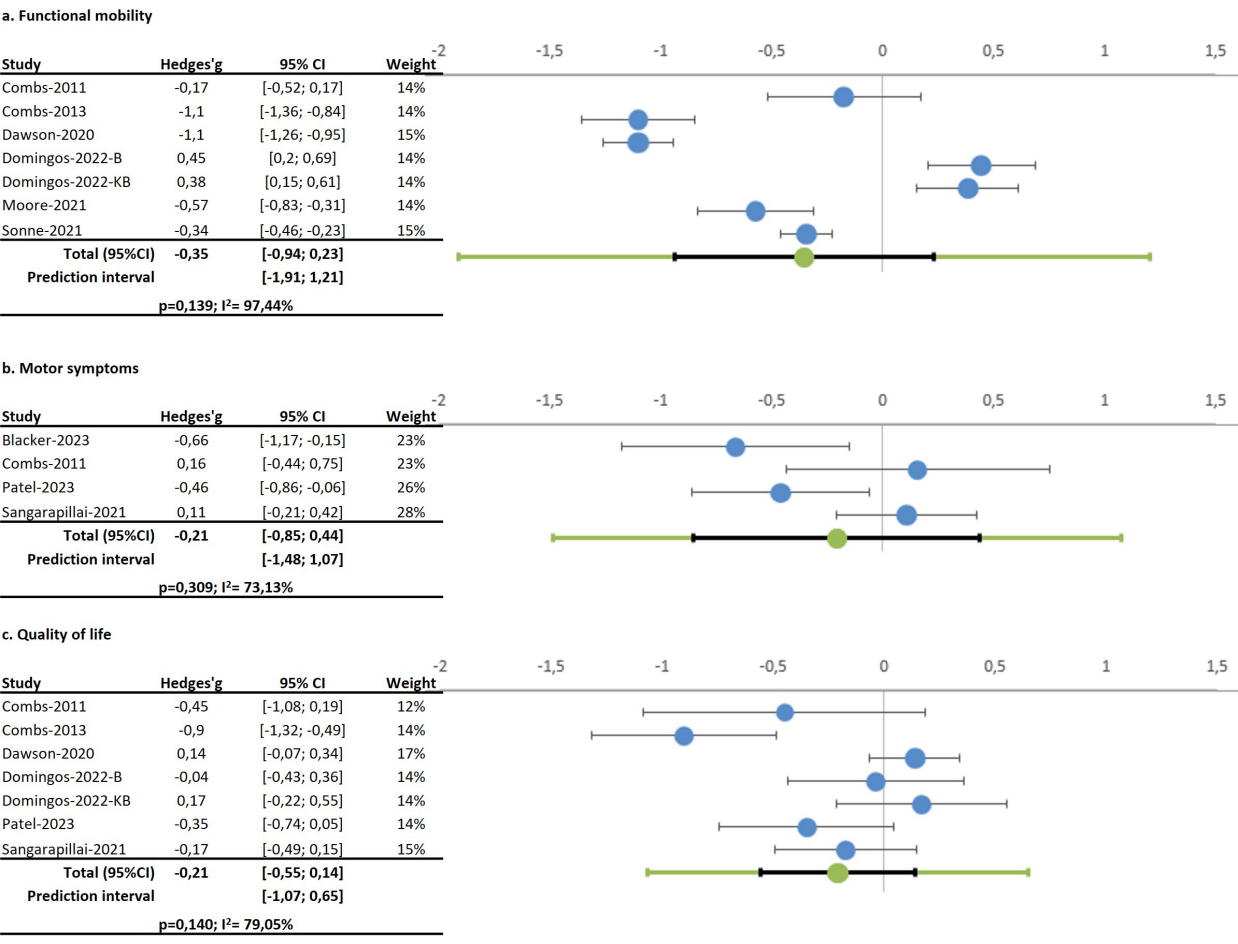
**Forest plot on the effects of Boxing on (a) Functional mobility, (b) Motor symptoms, (c) Quality of life**.

Six studies assessed boxing’s effects on balance, with mixed results [16, 18, 24, 25, 26, 28]. While some reported slight improvements [28], others noted reductions in balance levels [16, 24, 25]. Overall, pooled data indicated no significant changes in balance (Hedges’ g 0.06; 95% CI –0.39; 0.50, *p = *0.742), with high heterogeneity (I^2^ = 89.95%) and no publication bias (Egger’s *p* = 0.732). See Fig. 2.

#### 4.1.3 Motor Symptoms 

Four studies evaluated boxing’s effects on motor symptoms via the Unified Parkinson Disease Rating Scale part III (UPDRS-III), showing mixed results. While two studies reported significant positive effects [27, 28], one indicated worsened symptoms, and another found no improvements [27]. Pooled data showed no significant impact (Hedges’ g –0.21; 95% CI –0.85; 0.44, *p* = 0.309), with high heterogeneity (I^2^ = 76.13%).

#### 4.1.4 Gait

**Fig. 3.**
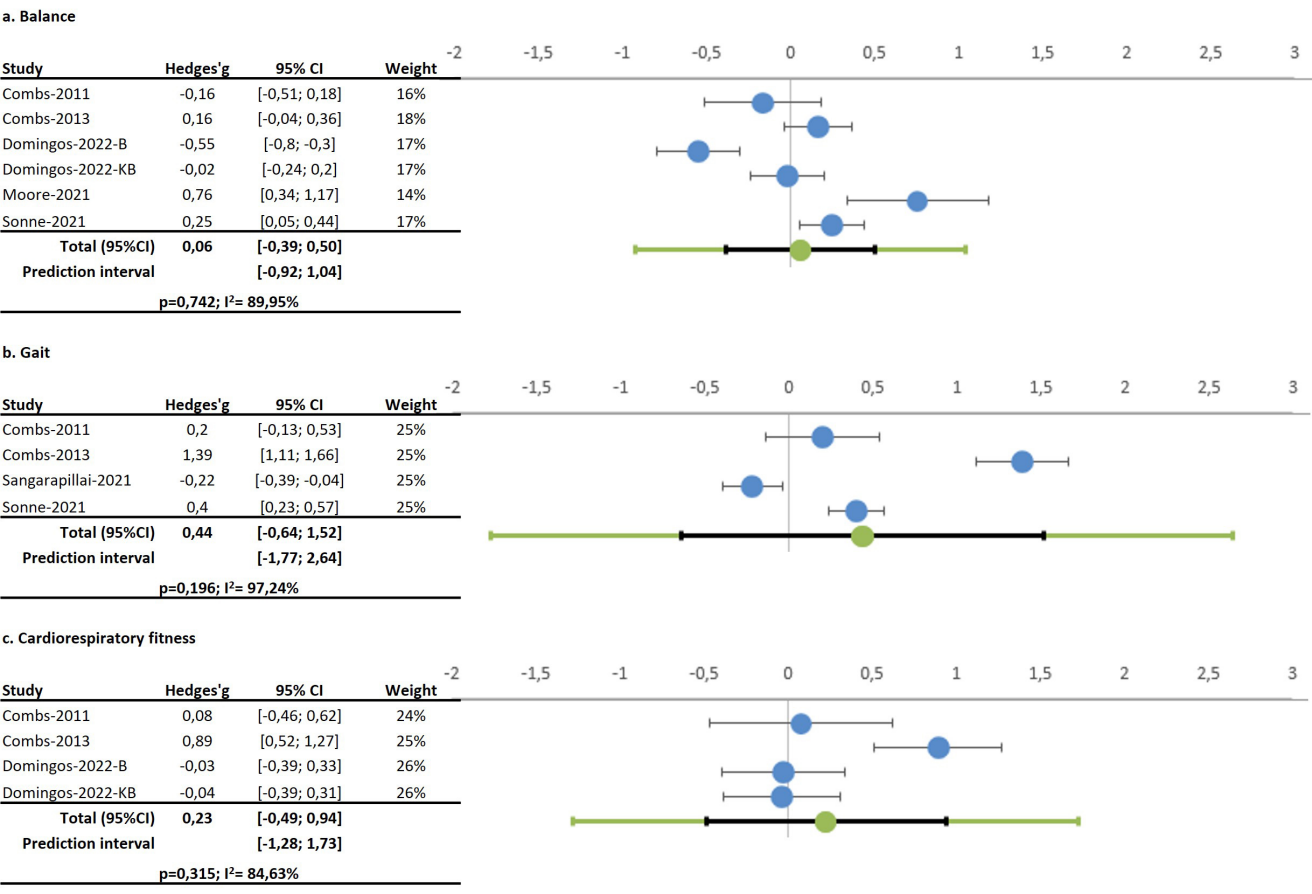
**Forest plot on the effects of Boxing on (a) Balance, (b) Gait and (c) Cardiorespiratory fitness**.

Four studies examined boxing’s association with gait parameters [17, 18, 25, 28]. Improvements in gait speed [18, 28], cadence and stride were noted [28], but some studies reported negative impacts on stride length [17]. Pooled data revealed no significant effect on gait (Hedges’ g 0.44; 95% CI –0.64; 1.52, *p = *0.196), with high heterogeneity (I^2^ = 97.24%). Comparison between experimental and control groups also showed non-significant results [17, 18] (Hedges’ g 0.31; 95% CI –2.36; 2.99, *p* = 0.136). A low heterogeneity was found (I^2^ = 0%), as shown in Fig. 3.

#### 4.1.5 Cardiorespiratory Fitness 

Four studies using the six-minute walk test (6MWT) indicated mixed results. While some studies found boxing to significantly improve fitness [18, 28], others did not report changes [16, 19]. Pooled data indicated no significant effect on cardiorespiratory fitness (Hedges’ g 0.23; 95% CI –0.49; 0.94, *p = *0.315), with high heterogeneity (I^2^ = 84.63%).

#### 4.1.6 Respiratory and Phonation 

One study showed significant improvements in maximum expiratory pressure [21], but no effects on transglottal airflow or subglottal pressure.

#### 4.1.7 Fear of Falling 

Boxing had no significant impact on fear of falling, according to the sole study that investigated this outcome [16].

### 4.2 Mental Health

#### 4.2.1 Quality of Life 

Seven studies assessed the impact of boxing on self-reported quality of life (QoL) using various questionnaires, with five showing significant benefits [16, 18, 22, 23, 28]. However, Sangarapillai *et al*. [17] and Patel *et al*. [27] found no significant changes, and Dawson *et al*. [23] reported a slight increase in pain. Pooled data from seven interventions [16, 18, 22, 23, 28] (n = 131) indicated a non-significant trend toward improved QoL (Hedges’ g –0.21; 95% CI –0.55; 0.14, *p* = 0.140), with high heterogeneity (I^2^ = 79.05%) and possible publication bias (Egger’s *p* = 0.104). Comparisons between experimental and control groups [17, 18] also yielded non-significant results (Hedges’ g 0.08; 95% CI –0.68; 0.83, *p* = 0.186).

#### 4.2.2 Depression

Boxing significantly reduced depression levels, as reported in two studies using the Movement Disorders Society Non-Motor Rating Scale (MDS-NMS) and the Hamilton Depression Scale (HDS) [27]. Patel *et al*. [27] observed significant reductions in the HDS for community-based boxing, while Urrutia *et al*. [22] noted improvements in the HDS score after a 12-week high-intensity boxing program, with significant improvements in sleep quality as well.

### 4.3 Sleep Disturbances 

One study indicated that high-intensity boxing led to significant improvements in sleep disturbances, assessed using the Parkinson Disease Sleep Scale [22]. 

### 4.4 Executive Function

One study found that participants in a non-contact boxing program significantly reduced their completion time for the Trail Making Test A compared to the control group [19].

### 4.5 Dropouts and Adverse Events 

Across seven studies reporting dropouts, a total of 42 participants withdrew [16, 18, 19, 22, 23, 27, 28]. Only one study reported adverse events related to boxing practice (n = 4) [20].

### 4.6 Methodological Quality 

The methodological quality of the three reviewed randomized controlled trials (RCTs) was rated as “good” [16, 17, 18]. Non-randomized studies typically utilized appropriate measurements for assessing outcomes and interventions, with most providing complete outcome data. However, none controlled for confounders in their design and analysis, and only two studies included participants representative of the target population [20, 27] (Table 2, Ref. [16, 17, 18, 19, 20, 21, 22, 23, 24, 25, 26, 27, 28]).

**Table 2.** **Methodological quality appraisal of the included studies**.

Study	Assessment items										
Randomized controlled trials (Physiotherapy Evidence Database, PEDro scale)	Random allocation	Concealed allocation	Baseline comparability	Blind subjects	Blind therapists	Blind assessors	Adequate follow-up	Intention-to-treat analysis	Between-group comparisons	Point estimates and variability	Total Score
Domingos *et al*. (2022) [16]	+	-	+	-	-	+	+	+	+	+	7/10
Sangarapillai *et al*. (2021) [17]	+	-	+	-	-	+	+	+	+	+	7/10
Combs *et al*. (2013) [18]	+	+	+	-	-	+	-	+	+	+	7/10
Non-randomized trials (Mixed Methods Appraisal Tool, MMAT)	1. Participants representative of target population		2. Appropriate measurements of outcome and intervention/exposure		3. Complete outcome data		4. Confounders accounted for in design and analysis		5. Intervention administered (or exposure occurred) as intended		
Blacker *et al*. (2023) [20]	+		+		+		-		+		
Combs *et al*. (2011) [28]	-		+		+		-		-		
Dawson *et al*. (2020) [23]	-		+		-		-		-		
Horbinski *et al*. (2021) [26]	-		-		+		-		-		
Moore *et al*. (2021) [24]	-		+		+		-		-		
Patel *et al*. (2023) [27]	+		+		+		-		-		
Salvatore *et al*. (2022) [19]	-		+		+		-		-		
Sonne *et al*. (2021) [25]	-		+		-		-		-		
Urrutia *et al*. (2020) [22]	-		+		-		-		+		
Watts *et al*. (2023) [21]	-		+		+		*-*		*-*		

## 5. Discussion

This systematic review aimed to determine if boxing is an effective rehabilitation strategy for patients with Parkinson’s Disease (PD). We included all experimental study designs, not just randomized controlled trials (RCTs) [29, 30]. The studies reviewed generally exhibited acceptable methodological quality, allowing for discussions on factors that could enhance the understanding of boxing’s potential benefits for PD patients.

Decreased movement ability is a key hallmark of PD, associated with a higher risk of lifestyle-related diseases and inadequate disease management [31]. Furthermore, critical aspects of mobility impairments in PD patients are often unresponsive to pharmacological or surgical therapies [32], emphasizing the need for alternative rehabilitation methods.

We examined evidence regarding boxing’s effects on outcomes like functional mobility, balance, and gait. Results were mixed, with some studies showing significant improvements while others found no significant effects, suggesting that traditional exercises might yield better outcomes [33, 34]. The meta-analysis failed to demonstrate significant positive effects of boxing on these measures, indicating that boxing might not be suitable for improving gait [35], a common and debilitating symptom of PD [36].

For motor symptoms, while various exercise modalities have shown benefits (3), boxing did not produce significant improvements, as indicated by the pooled data. This lack of effect is crucial for clinicians who need evidence to guide exercise prescriptions for PD motor symptoms [37]. Additionally, boxing did not lead to improvements in cardiorespiratory function, which might be due to inadequate intensity in the programs to sufficiently stimulate aerobic fitness [38].

Regarding mental health, particularly quality of life (QoL) and depression, previous research indicates exercise’s positive effects on these outcomes [39]. While some studies initially showed promising findings for QoL, the meta-analysis revealed no significant improvements. Regarding depression, preliminary evidence was positive, but limited studies prevented a comprehensive analysis.

Boxing did demonstrate some benefits in areas like sleep disturbances and executive function, yet the limited number of studies restricts further discussion. Mild adverse effects were reported, raising concerns about feasibility and safety for PD patients.

Several methodological weaknesses limit the applicability of these results. Not all studies included comparison groups, and many had small sample sizes, short durations, and lacked follow-up phases. Additionally, the inclusion of heterogeneous studies in the meta-analysis complicates the findings. The participants were primarily individuals with mild to moderate PD, questioning the generalizability of the results to those with more severe conditions. Limitations related to language and the exclusion of grey literature should also be noted.

## 6. Conclusions 

In conclusion, evidence regarding boxing as a prescription for PD patients remains uncertain. While preliminary findings suggest a lack of significant impact on physical and mental outcomes, limitations in the reviewed studies necessitate further research with larger sample sizes and more rigorous methodologies to clarify the potential benefits of boxing for individuals with PD.

## Availability of Data and Materials

No data were generated in this manuscript, the data used are available in public databases.

## Supplementary Material

Supplementary material associated with this article can be found, in the online version, at https://doi.org/10.31083/RN36478.
